# Supplementary material for: Overactive bladder and bladder pain syndrome/interstitial cystitis in primary Sjögren’s syndrome patients: A nationwide population-based study
Source: PLoS One. 2019 Nov 20;14(11):e0225455. doi: 10.1371/journal.pone.0225455 (PMC6867625; doi:10.1371/journal.pone.0225455)
Supplement: Attachment — Submitted filename: Rebuttal letter.doc [file pone.0225455.s001.doc]

# Reply to the comments and suggestions from Reviewer 1

**Thanks for your excellent review and useful comments**

I commend the authors on a well written manuscript on an interesting finding. The high incidence of OAB and BPS/IC in women with Sjogren's is remarkable for sure. I have several questions/comments for the authors:

1) Under 'Data Source' line 5 should read " Taiwan are included"

We corrected the typo in the method section (page 5, line 5).

2) Can you further explain the decision to exclude subjects with diagnosis before 2001? Was the decision for newly diagnosed patients only to see progression more objectively?

In the Longitudinal Health Insurance Database (LHID) used in our study, missing data existed more frequently before 2000. Moreover, in order to exactly identify the index date to further analysis of hazard ratio for OAB and BPS/IC, we only enrolled newly diagnosed patients with primary Sjögren’s syndrome. We revised our method section (page 5, lines 19-20)

3) In "Risks of OAB and BPS/IC" Why was the age of 65 years old chosen as a cut off?

The chronological age of 65 years is widely accepted as a definition of elderly in developed countries by many organizations including World Health Organization. Since Sjogren’s syndrome is an autoimmune disease involving female aged fifties to sixties and the mean age of pSS patients is our study was 53.8 ± 14.2 years, we decided to dichotomize age groups by 65 years in the analysis.

4) In "Risks of OAB and BPS/IC" lines 12-14 does not make sense. I am not sure what you are trying to say here. Please rewrite.

We revised our result section (page 9, lines 13-14)

5) In "Discussion" Why do you think rates of OAB BPS/IC were lower in your normal cohort population than described rates? Do you think it could be due to under diagnosis by physicians in the care of "normal" patients? Please explains.

Previous studies of OAB and BPS/IC prevalence rates defined their cases mainly by questionnaires, while our study was based on claims database. We agreed with the reviewer’s opinion that there might be an underdiagnosis of OAB or BPS/IC by physicians in the non-pSS group. We revised our discussion section accordingly (page17, lines 1-4)

Thank you for your insightful suggestions.

# Reply to the comments and suggestions from Reviewer 2

**Thanks for your excellent review and useful comments**

Sjögren’s syndrome (SS) is an autoimmune disease affecting lacrimal and salivary glands. Primary SS has long been associated with interstitial cystitis/bladder pain syndrome (IC), but the extent of association remains unclear. Lee and colleagues used data from Taiwan’s National Health Insurance Research Database to examine the occurrence of IC and overactive bladder (OAB) among newly-diagnosed pSS patients over a 10-year period, relative to a non-pSS control group. Among 11,526 pSS patients, elevated hazard ratios were identified for both IC and OAB, and younger age, female gender, and sever glandular dysfunction were identified as risk factors. The manuscript is generally well written, although tables should not be incorporated into the text, and page numbers would facilitate review.

Concerns: 1. On Page 15, minor editing should be applied to Lines 5-8. “…mast cells may induce…” and “drive mast cells to the bladder lamina propria…”

Thanks for your valuable comments. We added page numbers to the manuscript and revised our discussion section. (page 15, lines 5 and 7)
